# Complement-Mediated Thrombotic Microangiopathy in Pregnancy: An Educational Case Report

**DOI:** 10.1177/20543581231209009

**Published:** 2023-11-06

**Authors:** Valentina Bruno, David Barth, Arenn Jauhal

**Affiliations:** 1 Division of Nephrology, Department of Pediatrics, The Hospital for Sick Children, University of Toronto, ON, Canada; 2 Cell Biology Program, SickKids Research Institute, The Hospital for Sick Children, University of Toronto, ON, Canada; 3 Division of Medical Oncology and Hematology, University Health Network, Temerty Faculty of Medicine, University of Toronto, ON, Canada; 4 Departments of Medicine and Laboratory Medicine & Pathobiology, University of Toronto, ON, Canada; 5 Division of Nephrology, Department of Medicine, Glomerulonephritis Clinic, Toronto General Hospital, University Health Network, Temerty Faculty of Medicine, University of Toronto, ON, Canada

**Keywords:** atypical hemolytic uremic syndrome, pregnancy, complement system, C5 inhibitor

## Abstract

**Rationale:**

Thrombotic microangiopathy (TMA) is a spectrum of rare diseases characterized by thrombocytopenia, microangiopathic hemolytic anemia, and organ damage. Differentiating pre-eclampsia, HELLP (Hemolysis, Elevated Liver enzymes, Low Platelets) syndrome and atypical hemolytic uremic syndrome (aHUS) during pregnancy may be diagnostically challenging yet important as the treatment pathways differ. Most cases of aHUS are associated with dysregulation of the complement alternative pathway, for which current guidelines recommend prompt treatment with complement C5 inhibitor to prevent chronic sequelae. Here, we report a case of pregnancy-associated aHUS (p-aHUS) to highlight the challenging aspects of the diagnostic process and the importance of prompt treatment with complement inhibition to reduce the risk of poor outcomes.

**Presenting concerns:**

A 28-year-old woman was admitted to a local hospital for induction of vaginal delivery of twins at 34 weeks and 3 days of gestational age, due to intrauterine growth restriction (IUGR). She was previously healthy, and this current pregnancy was uncomplicated, except for the IUGR. Approximately, 10 hours after her induced delivery, she developed vomiting, epigastric pain, and hypertension.

**Diagnosis:**

She was initially suspected of having fulminant liver failure in the context of acute fatty liver of pregnancy versus pre-eclampsia/HELLP syndrome, due to evidence of elevated liver enzymes, acute kidney injury (AKI), thrombocytopenia, and hemoglobin levels trending down, for which the patient was initially treated conservatively. On day 2 post-delivery, she was transferred to our hospital for possible liver biopsy and management of liver failure. Upon transfer, dialysis was started due to anuric AKI; at the same time, her liver function spontaneously improved, while platelet count remained very low and hemoglobin levels continued to trend down. A full TMA work-up revealed low C3 levels; secondary causes of TMA were ruled out. The patient received a final diagnosis of p-aHUS. Complement genetic tests were also performed and did not identify any pathogenic variants.

**Interventions:**

Given the final diagnosis of p-aHUS, the patient was started on a C5 inhibitor (day 8 post-delivery). Her platelet count quickly normalized 2 days after the first dose, while the hemoglobin levels remained low for a longer period, likely due to retained products of conception.

**Outcomes:**

The patient was able to completely discontinue dialysis after approximately 3 months, however, her kidney function did not recover completely, despite all the other TMA markers normalizing (platelets count in range, negative hemolysis markers, and normal hemoglobin levels). Her estimated glomerular filtration rate (eGFR) was 23 mL/min/1.73 m^2^ at the 6-month follow-up.

**Teaching points:**

The diagnosis of p-aHUS can be challenging due to frequent overlapping symptoms and signs with other forms of pregnancy-associated TMA, leading to a delay of the treatment, which can affect the patient’s outcome. Failure of TMA to improve in the postpartum period or occurring at this time, with negative ADAMTS13 and antiphospholipid antibody syndrome (APLAS) serologies should favor the diagnosis of p-aHUS. Early treatment with C5 inhibition should be considered in women with a diagnosis of p-aHUS. Patients need multidisciplinary and likely tertiary/quaternary care at centers where clinical experience, access to diagnostics and treatment initiation can begin without delay.

## Abrégé

**Justification:**

La microangiopathie thrombotique (MAT) est un spectre de maladies rares caractérisées par une thrombocytopénie, une anémie hémolytique microangiopathique et des lésions à différents organes. Pendant la grossesse, il peut être difficile de différencier la prééclampsie, le syndrome HELLP (Haemolysis, Elevated Liver enzymes, Low Platelets) et le syndrome hémolytique et urémique atypique (SHUa) d’un point de vue diagnostique, mais il est important de le faire, car les avenues de traitement diffèrent. La plupart des cas de SHUa sont associés à un dérèglement de la voie alterne du complément, pour lequel la recommandation actuelle est de privilégier un traitement rapide avec un inhibiteur de la fraction C5 du complément afin de prévenir les séquelles chroniques. Dans le présent article, nous présentons un cas de SHUa associé à une grossesse (SHUa-g) afin de mettre en évidence les difficultés associées au diagnostic et l’importance de traiter rapidement par inhibition du complément afin de réduire le risque de résultats défavorables.

**Présentation du cas:**

Une femme de 28 ans admise dans un hôpital local pour l’induction d’un accouchement vaginal de jumeaux à 34 semaines et 3 jours, en raison d’un retard de croissance intra-utérin (RCIU). La patiente était en bonne santé et la grossesse, à l’exception du RCIU, était sans complications. Environ dix heures après l’accouchement, la patiente a développé des vomissements, des douleurs épigastriques et de l’hypertension.

**Diagnostic:**

On a d’abord soupçonné une insuffisance hépatique fulminante liée soit à une stéatose hépatique aiguë de la grossesse ou encore à une prééclampsie/syndrome HELLP, en raison d’une élévation des enzymes hépatiques, d’une insuffisance rénale aiguë, d’une thrombocytopénie et d’une baisse de l’hémoglobine, pour lesquelles la patiente a initialement été traitée de façon conservatrice. Deux jours après l’accouchement, la patiente a été transférée à notre hôpital pour une possible biopsie du foie et la prise en charge d’une insuffisance hépatique. Après le transfert, une IRA anurique a justifié l’initiation de la dialyse. Au même moment, la fonction hépatique s’est améliorée spontanément, tandis que la numération plaquettaire est demeurée très faible et que les taux d’hémoglobine ont continué à baisser. Un bilan MAT complet a révélé de faibles taux de C3; les causes secondaires de MAT ont été écartées. La patiente a reçu un diagnostic final de SHUa-g. Des tests génétiques complémentaires n’ont pas permis d’identifier de variants pathogènes.

**Intervention:**

Compte tenu du diagnostic final de SHUa-g, un traitement avec un inhibiteur de C5 a été amorcé (au huitième jour après l’accouchement). La numération plaquettaire s’est rapidement normalisée deux jours après la première dose, mais les taux d’hémoglobine sont demeurés bas sur une plus longue période, probablement en raison de la rétention de produits de la conception.

**Résultats:**

La patiente a pu cesser complètement la dialyse environ trois mois plus tard, mais sa fonction rénale ne s’est pas complètement rétablie, malgré la normalisation de tous les autres marqueurs de MAT (numération plaquettaire dans les normales, marqueurs d’hémolyse négatifs, taux d’hémoglobine normaux). Son débit de filtration glomérulaire estimé (DFGe) s’établissait à 23 ml/min/1.73 m^2^ lors du suivi à six mois.

**Enseignements tirés:**

Le chevauchement fréquent des symptômes et signes cliniques des autres formes de MAT associées à la grossesse rend parfois difficile le diagnostic du SHUa-g, ce qui peut retarder le traitement et affecter l’évolution clinique. L’absence d’amélioration d’une MAT dans la période postpartum, ou sa survenue à ce moment, couplée à des sérologies négatives pour ADAMTS13 et pour le syndrome des anticorps antiphospholipides (SAPL) devrait plaider en faveur d’un diagnostic de SHUa-g. Un traitement précoce par inhibition de C5 doit être envisagé chez les femmes qui reçoivent un diagnostic de SHUa-g. Ces patientes requièrent des soins multidisciplinaires, et probablement tertiaires/quaternaires, dans des centres où l’expérience clinique, l’accès au diagnostic et l’initiation du traitement sont rapidement accessibles.

## Introduction

Atypical hemolytic uremic syndrome (aHUS) is a rare form of thrombotic microangiopathy (TMA) not associated with Shiga toxin-producing *Escherichia coli* (STEC) infection, which can be triggered by other infections (especially in children), autoimmune conditions, malignancy, organ transplantation, pregnancy, or drugs. It is characterized by the classical TMA triad, that is, thrombocytopenia, microangiopathic hemolytic anemia and organ (especially kidney) damage, and in most cases, it is associated with dysregulation of the complement alternative pathway (CAP).^1^

The incidence of aHUS is approximately 0.5 per 1 million people, however, this can vary by population.^2^ Interestingly, 10% to 20% of aHUS cases are diagnosed in the context of pregnancy, defined as pregnancy-associated aHUS (p-aHUS).^3^

A normal pregnancy relies on a tight regulation of the complement system, as suggested by the fact that hyperactivation and/or dysregulation of the CAP can lead to several pregnancy complications, such as pre-eclampsia/HELLP (Hemolysis, Elevated Liver enzymes, Low Platelets) syndrome and p-aHUS.^4,5^

Pregnancy-associated aHUS is a rare form of TMA, triggered by pregnancy, requiring prompt diagnosis and therapy, as delayed treatment is associated with significant perinatal and/or maternal morbidity and mortality. Interestingly, both p-aHUS and complement-mediated aHUS are characterized by severe renal involvement, with 40% to 70% of patients requiring dialysis, and poor renal outcomes, as approximately 53% of cases develop end-stage kidney disease in absence of specific treatments.^3^ Moreover, both conditions are associated with similar complement gene variants in 40% to 56% of cases.^6,7^ For this reason, p-aHUS is currently considered as part of the spectrum of complement-mediated aHUS.

The diagnosis of p-aHUS remains challenging, as both clinical and laboratory characteristics can overlap with other pregnancy complications, such as pre-eclampsia/HELLP syndrome. Furthermore, despite growing evidence suggesting that complement system plays a role in p-aHUS,^8,9^ the use of anti-complement therapies is not well established in this condition.

## Presenting Concerns

A 28-year-old woman, previously healthy, was admitted to a local hospital for induction of vaginal delivery of twins due to intrauterine growth restriction (IUGR).

She delivered dichorionic diamniotic twins via induced vaginal birth at 34 weeks and 3 days of gestational age with no complications. Approximately, 10 hours after her delivery, she developed vomiting, epigastric pain, and hypertension.

## Clinical Findings

### Medical History

She was primigravida and no additional complications were reported during her pregnancy, except for the IUGR. Given the twin pregnancy, her obstetrician prescribed aspirin for risk reduction of pre-eclampsia. She did not report any recent history of diarrhea or loose bowel motions. Her past medical history was unremarkable, except for 1 episode of supraventricular tachycardia 5 years before, for which she was treated with bisoprolol for approximately 2 years.

On admission, she was on acetylsalicylic acid 162 mg daily, ferrous fumarate 300 mg daily, cholecalciferol 1000 units daily, and multiple vitamin supplements.

### Physical Examination and Diagnostic Testing

On clinical assessment, she appeared drowsy. She was afebrile. There was diffuse peripheral edema and hypertension, with a blood pressure of 144/103 mm Hg. No headache or visual changes were reported. No chest pain and/or shortness of breath.

Moreover, there was mild tenderness in the right upper quadrant of the abdomen with deep palpation.

Her laboratory investigations, approximately 12 to 18 hours after her delivery, showed thrombocytopenia (platelet count dropped to 72 × 10^9^/L from a baseline of 246 × 10^9^/L), elevated liver enzymes (aspartate transaminase [AST], 298 UI/L; alanine transaminase [ALT], 401 UI/L; alkaline phosphatase [ALP], 319 UI/L), increased bilirubin levels (total bilirubin, 111 μmol/L; direct bilirubin, 77 μmol/L), elevated lactate dehydrogenase (LDH) at 1684 UI/L, activated partial thromboplastin time (aPTT) 42 seconds, INR 2.1, hemoglobin levels at 143 g/L, white blood cell count at 31.2 × 10^9^/L, serum glucose, 3.5 mmol/L.

From a renal perspective, she developed an acute kidney injury (AKI) and metabolic acidosis (serum creatinine increased to 280 μmol/L from a baseline of 45 μmol/L, blood urea nitrogen 14.2 mmol/L, serum sodium 132 mmol/L, serum potassium 5.2 mmol/L, and serum bicarbonate 19 mmol/L). Urine protein-creatinine ratio was 103 mg/mmol.

An abdominal x-ray was performed, and it was initially concerning for possible ileus, however, this was not confirmed by computed tomography (CT) abdominal scan. The CT revealed kidneys that were unremarkable in appearance, with each measuring approximately 9.5 cm and no evidence of hydronephrosis.

## Diagnostic Focus and Assessment

Internal medicine, gastroenterology, and nephrology teams of her local hospital were consulted and they suspected fulminant liver failure in the context of acute fatty liver of pregnancy (AFLP) versus pre-eclampsia/HELLP syndrome, for which the patient was transferred to intensive care unit (ICU) and initially managed with labetalol infusion, intravenous magnesium supplementation, and vitamin K. She also received intravenous hydration to manage a possible pre-renal component of her AKI in the context of persistent vomiting and dehydration. Non-steroidal anti-inflammatory drugs, previously used to manage her pain, were also discontinued. Moreover, she was started on empirical piperacillin-tazobactam while waiting for the results of her blood cultures, later stopped as soon as the results came back negative. While admitted in ICU, her laboratory tests were monitored as summarized in Table 1. An abdomen ultrasound was also performed and showed only mildly increased echogenicity of the liver parenchyma, suggesting mild hepatic steatosis, and trace of free fluid.

**Table 1.** Main Laboratory Investigations Pre-Delivery (34 Weeks of GA, Gestational Age) and Post-Delivery (Day 1 and Day 2).

	Pre-delivery (34 weeks GA)	Day 1 post 5:00 pm	Day 1 post 09:00 pm	Day 2 post 05:30 am	Day 2 post 12:30 pm
Hemoglobin (g/L)	143	143	131	129	128
Platelet count (× 10^9^/L)	246	72	61	54	59
aPTT (seconds)	—	—	42	40.9	35.4
INR	—	—	2.1	2.0	—
AST (UI/L)	—	—	298	389	368
ALT (UI/L)	—	—	401	395	356
ALP (UI/L)	—	—	319	340	346
LDH (UI/L)	—	—	1684	—	—
Total bilirubin (μmol/L)	—	—	111	114	139
Direct bilirubin (μmol/L)	—	—	77	79	94
Serum creatinine (μmol/L)	45	280	318	385	455
Blood urea nitrogen (mmol/L)	—	—	14.2	17.3	18.3
Serum sodium (mmol/L)	—	132	132	130	130
Serum potassium (mmol/L)	—	5.2	5.5	5.3	5.4
Serum albumin (g/L)	—	—	—	—	20

*Note.* GA = gestational age; aPTT = activated partial thromboplastin time; INR = international normalized ratio; AST = aspartate transaminase; ALT = alanine transaminase; ALP = alkaline phosphatase; LDH = lactate dehydrogenase.

On day 2 post-delivery, given the rapid progression of her liver failure, she was transferred to the ICU of our hospital for close monitoring and consideration of liver transplant.

Unfortunately, the patient continued to have diffuse severe peripheral edema and she became completely anuric in the context of worsening kidney function, with signs of pulmonary edema on her chest X-ray, requiring to start renal replacement therapy on the same day of her transfer to our hospital. She received sustained low-efficiency daily dialysis (SLEDD) for 2 days, followed by intermittent hemodialysis. After initiation of dialysis, blood pressure improved to approximately 130/80 mm Hg and remained in this range.

She did not show signs of encephalopathy and her liver function rapidly improved within 72 hours post-delivery, while having persistent AKI (serum creatinine peak of 474 μmol/L, pre-dialysis) requiring renal replacement therapy, thrombocytopenia (platelet count worsened to 31 × 10^9^/L), ongoing markers of hemolysis (LDH peak of 2840 UI/L, haptoglobin levels undetectable, presence of schistocytes on the peripheral blood smear), and progressive anemia (hemoglobin levels gradually reduced from 143 to 73 g/L).

Given both her clinical course and the trend of her laboratory investigations, the ongoing diagnosis of AFLP versus HELLP syndrome became less likely and the patient underwent a complete TMA assessment, including autoimmune and infectious work-up, complement levels, and ADAMTS13 activity as recommended by both nephrology and hematology. However, given the early improvement in liver enzymes, one possibility was early pre-eclampsia/HELLP that resolved after delivery, but precipitated a complement-mediated TMA process.

The TMA work-up showed reduction of the C3 levels at 0.55 g/L (normal value = 0.98-1.96 g/L), with normal C4 levels. There was no evidence of secondary causes of TMA. Thrombotic thrombocytopenic purpura (TTP) was also ruled out, as ADAMTS13 activity resulted normal. Plasmapheresis was not considered given the PLASMIC score did not indicate high risk, and the relatively quick turnaround time for ADAMTS13 activity.

At this point, the patient received the final diagnosis of p-aHUS. A genetic study was later performed (including the following genes: C3, C9, CD46, CFH, CFB, CFHR1, CFHR2, CFHR3, CFHR4, CFHR5, CFI, DGKE, F12, FKRP, INF2, MMACHC, MMADHC, PLG, ST3GAL1, THBD, and VWF), which did not identify any pathogenic variant in complement genes or other gene mutations associated with aHUS. Functional complement testing was not available at our center.

## Therapeutic Focus and Assessment

On day 8 post-delivery, after receiving anti-meningococcal vaccination and starting penicillin prophylaxis, the patient underwent treatment with eculizumab. She received 900 mg intravenous weekly for 4 doses, 1200 mg weekly for 1 dose, followed by 1200 mg every 2 weeks. Her platelet count progressively improved and returned to normal range 2 days after the first dose. The patient’s hemoglobin remained low for a longer period, which was thought to be secondary to retained products of conception that caused bleeding.

The patient was discharged home on day 24 post-delivery, on intermittent hemodialysis and maintenance dose of eculizumab (1200 mg intravenous every 2 weeks). On discharge, despite having normal platelet count (406 × 10^9^/L) and negative hemolysis markers, her hemoglobin levels continued to be low at 73 g/L and gradually improved only after undergoing a dilation and curettage (D&C) procedure for retained products of conception.

**Figure 1.**
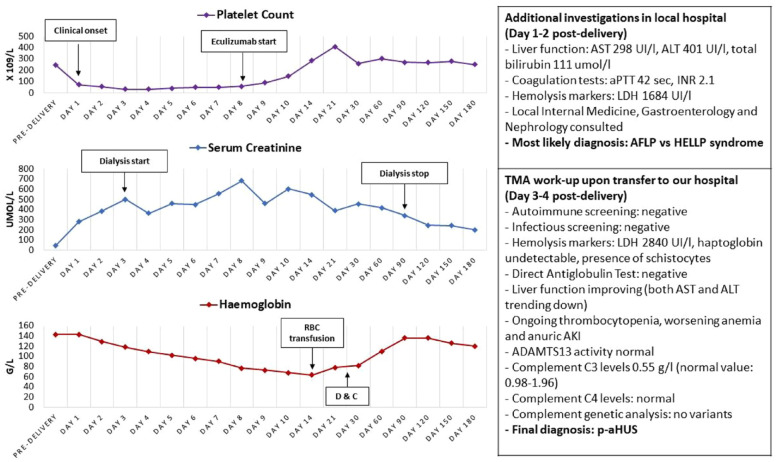
Timeline of clinical presentation, laboratory investigations, and treatment. *Note.* RBC = red blood cells; aPTT = activated partial thromboplastin time; INR = international normalized ratio; AST = aspartate transferase; ALT = alanine transaminase; ALP = alkaline phosphatase; LDH = lactate dehydrogenase; AFLP = acute fatty liver of pregnancy; HELLP = Hemolysis, Elevated Liver enzymes, Low Platelets; p-aHUS = pregnancy-associated atypical hemolytic uremic syndrome; D&C = dilation and curettage.

A timeline of clinical presentation, laboratory investigations, and therapeutic decisions is reported in Figure 1.

## Follow-up and Outcomes

After being discharged, the patient remained on C5 inhibition at the dose of 1200 mg every 2 weeks, with the plan to continue the treatment for total 12 months and then stop, while monitoring for recurrence of aHUS. Her serum CH50 levels continue to be undetectable, suggesting full complement inhibition. After 3 months, she was able to completely discontinue dialysis, however, her kidney function did not recover completely (serum creatinine = 200 μmol/L), despite persistent normalization of her hematological parameters (both platelet count and hemoglobin levels in normal range). Her eGFR remained low at 23 mL/min/1.73 m^2^ (serum creatinine = 243 μmol/L) by the end of 6-month follow-up.

## Discussion

The differential diagnosis of TMA during pregnancy or in the postpartum period (Table 2) can be particularly challenging. The most common forms of pregnancy-associated TMA are pregnancy-induced hypertension (PIH) and pre-eclampsia/HELLP syndrome.^10^ Less frequently, pregnancy-associated TMA can develop in the context of TTP or aHUS. Catastrophic antiphospholipid syndrome and systemic lupus erythematosus can be other causes of TMA during pregnancy or postpartum and, occasionally, AFLP can also present with TMA features.

**Table 2.** Main Forms of TMA in Pregnancy.

	Incidence/pregnancy	Low platelet count	Hypertension	Renal injury	Neurologic features	ADAMTS13 (UI/dL)
Pre-eclampsia	1/20	+ + +	+ + +	+/−	+ +	>20
HELLP syndrome	1/1000	+ + + +	+	+/−	+/−	>20
TTP	1/17 000-200 000	+ + ++	+	+	+ + +	<10
p-aHUS	1/25 000	+ + +	+ +	+ + +	+	>20

*Source.* Modified from Scully.^10^

*Note.* TMA = thrombotic microangiopathy; HELLP = Hemolysis, Elevated Liver enzymes, and Low Platelets; TTP = thrombotic thrombocytopenic purpura; p-aHUS = pregnancy-associated atypical hemolytic uremic syndrome.

Pregnancy-associated aHUS is a rare condition, representing 16% of all aHUS cases occurring in women aged 18 to 45 years. It tends to occur more frequently in the postpartum period (up to 3 months post-delivery). In particular, the onset of TMA in the postpartum of an uneventful pregnancy is considered very suggestive of p-aHUS.^8^

As mentioned in the “Introduction” section, p-aHUS is now considered part of the spectrum of complement-mediated aHUS and the use of C5 inhibition has been reported in more than 35 women with p-aHUS.^8^ In a retrospective study of 22 women diagnosed with p-HUS, 10 of 22 were treated with C5 inhibitor reaching both hematological and renal remission. Interestingly, none of the patients developed end-stage kidney disease by the end of 2-year follow-up.^6^ However, a possible publication bias needs to be considered while reviewing those cases, as only patients with successful outcome could have been reported in the literature. There have not been safety concerns reported with eculizumab use in pregnancy nor with breast feeding.^11^

We report the case of a 28-year-old woman who received the diagnosis of p-aHUS approximately 3 to 4 days after the onset of her symptoms, as the initial suspected diagnosis was AFLP versus HELLP syndrome. She received the first dose of eculizumab on day 8 post-delivery leading to a rapid normalization of her platelet count. Her kidney function improved and the patient was able to stop dialysis after 3 months. Given the high suspicion for TMA on renal histology, paired with the improvement in hematological parameters with C5 inhibition, a renal biopsy was not pursued given the low likelihood of alternative findings altering management.

Unfortunately, despite the recovery of her hematological parameters, her kidney function never normalized, while on maintenance dose of eculizumab every 2 weeks. Our plan is to continue this therapy for total 12 months, while monitoring serum CH50 levels to ensure full complement blockade, as well as her TMA markers to detect early signs of possible aHUS recurrence.

The early liver injury that resolved post-delivery was unclear, but the possibility of initial pre-eclampsia/HELLP syndrome remains. This may have improved after delivery and triggered a complement-mediated TMA.

Interestingly, compared with the cases of p-aHUS reported in literature, our patient did not have normalization of eGFR. This could be related to the delay in diagnosis and initiation of anti-complement therapy. The first dose of eculizumab was provided 8 days after the onset of her symptoms. The patient may continue to have ongoing improvement in serum creatinine through months 6 to 12.

From a diagnostic perspective, the approach to pregnancy-associated TMA requires an early and complete work-up to investigate for primary and secondary causes of TMA. Avoiding delay is imperative to help optimize the patient’s long-term outcomes.^12^

In particular, the diagnosis of TTP should be immediately ruled in/out due to the potential life-threatening complications. As the measurement of the ADAMTS13 activity is usually not available as “urgent testing” in all centers, the PLASMIC and French scores,^13,14^ despite not being validated in pregnancy, may help predicting ADAMTS13 deficiency by combining clinical and laboratory parameters (platelet count < 30 × 10^9^/L; serum creatinine < 200 μmol/L; INR < 1.3; median corpuscular volume < 86.5 fL; hemolysis markers).

Some authors suggest that the initial TMA work-up should also include the soluble fms-like tyrosine kinase-1/placental growth factor (sFlt-1/PlGF) ratio, which has been described as useful tool to rule in/out hypertensive complications of pregnancy.^15^ SFlt-1/PlGF ratio higher than 85, if <34 weeks of gestational age, and higher than 110, if > 34 weeks of gestational age, are considered strongly suggestive of pre-eclampsia/HELLP syndrome, while a ratio less than 38 would suggest an alternative diagnosis. As the sFlt-1/PlGF ratio is not regularly available as “urgent test” to guide the initial approach, this marker could become used retrospectively to clarify the etiology of pregnancy-associated TMA and to optimize the treatment.

To date, there is no specific test to confirm the diagnosis of p-aHUS, which remains a diagnosis of exclusion. Interestingly, despite evidence supporting dysregulation of the complement system in this condition, a complement work-up is not required for the diagnosis and/or management of p-aHUS, as normal complement levels and/or absence of complement gene mutations do not exclude the diagnosis. However, a complement analysis, including serum (+/– urine) measurement of complement proteins, antibody anti-factor H, and genetic testing to detect possible pathogenic complement variants is still recommended in the initial phase, as this could change the short-term and/or long-term management, and rule in the diagnosis p-aHUS. For example, the use of immunosuppressive therapy, such as Rituximab, would be considered in case of anti-factor H antibodies; moreover, the identification of a pathogenic variant in 1 or more complement genes would confirm the diagnosis of complement-mediated HUS retrospectively, and it would be helpful to decide whether to continue long-term therapy with C5 inhibitor, as well as to predict possible risk of aHUS recurrence in future pregnancies.^16^ Unfortunately, anti-factor H antibodies were not available at our center.

To conclude, the prognosis of p-aHUS has dramatically improved since the advent of the anti-C5 therapy, which has been reported to be safe for both mother and fetus during pregnancy. When TMA fails to improve postpartum, with negative TTP and antiphospholipid antibody syndrome (APLAS) serologies, clinicians should highly suspect p-aHUS; the addition of abnormal complement serologies should strengthen this suspicion. A successful outcome relies on multiple aspects, including a prompt diagnosis leading to an early start of the anti-complement therapy, as well as a strict therapeutic monitoring to assess the degree of terminal complement blockade.
